# Left-Sided Intra-thoracic Ectopic Kidney With Symptomatic Bochdalek Hernia: A Case Report

**DOI:** 10.7759/cureus.65452

**Published:** 2024-07-26

**Authors:** Manasa P, Isha Shah, Shiyam Sundaran P, G Murugan

**Affiliations:** 1 Department of Radiology, Sree Balaji Medical College and Hospital, Chennai, IND

**Keywords:** ct urography, congenital diaphragmatic hernia, intra-thoracic kidney, ectopic kidney, bochdalek hernia

## Abstract

A congenital defect in the diaphragm, known as a Bochdalek hernia (BH), is a condition that allows herniation of the abdominal viscera into the thorax. BH is the most common type of congenital diaphragmatic hernia (CDH) and is typically detected on the left side. An ectopic kidney is a rare condition. An intra-thoracic ectopic kidney is an extremely uncommon condition. In adult patients, the presence of BH with an intra-thoracic kidney is extremely uncommon and is often a finding discovered unintentionally. A 51-year-old male patient presented to the outpatient unit of the pulmonology department. He stated that he had been suffering symptoms such as coughing, wheezing, and breathing difficulties for one year. A chest X-ray showed a well-defined radio-opaque lesion in the lower left zone. A computed tomography (CT) scan of the chest demonstrated a defect in the posterolateral region of the left hemidiaphragm, as well as herniation of the left kidney and retroperitoneal fat in the left hemithorax. The intra-thoracic ectopic kidney was found to be normal in size and showed normal attenuation and enhancement, with the contrast being promptly excreted into the pelvicalyceal system during CT urography. Due to the hernia's small size and lack of abnormalities on CT urography, the patient was recommended a conservative treatment. A follow-up examination was performed on the patient annually. Throughout the follow-up period, there was not a single episode of kidney-related issues. To avoid unwanted image-guided biopsies and surgical procedures, it is imperative to look for intra-thoracic kidneys in patients presenting with a thoracic mass or an elevated hemi-diaphragm.

## Introduction

A congenital defect in the posterolateral aspect of the diaphragm, known as a Bochdalek hernia (BH), is a condition that allows herniation of the abdominal viscera into the thorax. BH is the most common type of congenital diaphragmatic hernia (CDH) and is typically detected on the left side. Concerning this type of CDH, approximately one in every 2,200 to 12,500 live births are affected [1]. An ectopic kidney is a rare phenomenon that occurs in approximately one in 1,000 live newborns. Intra-thoracic ectopic kidney is a very rare condition, making up less than 5% of all ectopic kidney cases [1-5]. Intra-thoracic renal ectopia is often detected as an incidental finding on chest X-ray, resembling a mass in the posterior mediastinum and requiring further evaluation [1,6,7]. The objective of the current case report is to present a detailed overview of a unique case involving an adult male patient who presented with a left-sided intra-thoracic kidney and Bochdalek hernia.

## Case presentation

**Figure 1:**
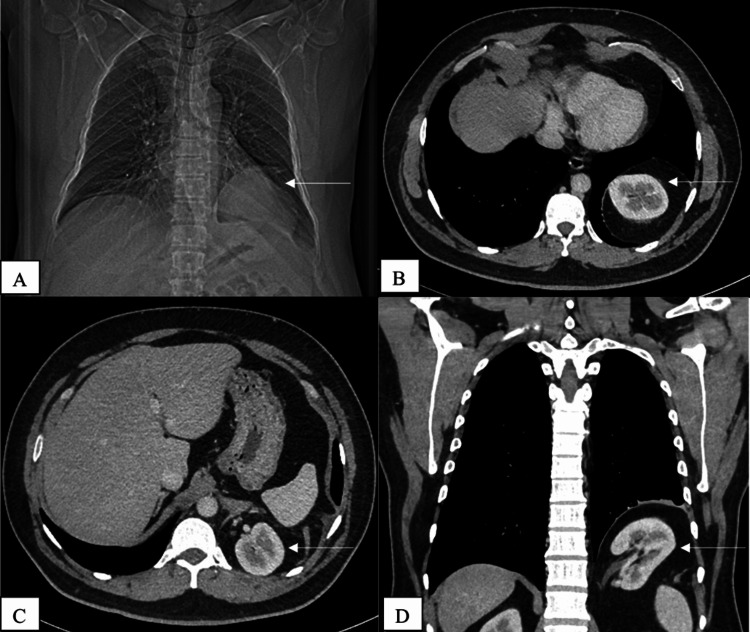
Bochdalek hernia with intra-thoracic kidney (A) Chest X-ray showing a well-defined radio-opaque lesion in the lower left zone (white arrow). (B) Axial view of contrast-enhanced computed tomography (CT) chest showing left intra-thoracic ectopic kidney (white arrow). Axial (C) and coronal (D) views of contrast-enhanced CT chest and abdomen showing an intra-thoracic kidney and Bochdalek hernia (white arrow).

A 51-year-old male patient presented to the outpatient unit of the pulmonology department. He stated that he had been suffering symptoms such as coughing, wheezing, and breathing difficulties for one year. Throughout the preceding five years, the patient had periodically reported experiencing identical symptoms. No symptoms such as a fever, chest pain, vomiting, loss of appetite, or weight loss had been reported. Upon initial examination, reduced breath sounds and rhonchi on both sides were detected on chest auscultation, and on percussion, dullness was present in the left lower zone. Standard diagnostic tests, such as complete blood counts and C-reactive protein were conducted, and all results remained in the normal range of values. The results of the sputum test for screening of* Mycobacterium tuberculosis* were negative. A chest X-ray showed a well-defined radio-opaque lesion in the lower left zone (Figure 1).

A chest computed tomography (CT) scan demonstrated a defect in the posterolateral region of the left hemidiaphragm, as well as herniation of the left kidney and retroperitoneal fat into the left hemithorax (Figure 1). The intra-thoracic ectopic kidney was found to be normal in size and showed normal attenuation and enhancement, with the contrast being promptly excreted into the pelvicalyceal system during the CT urography. The basal regions of the left lower lobe demonstrated atelectatic changes. There were no additional abnormalities observed in the thorax and abdomen. Due to hernia's smaller size and lack of abnormalities on CT urography, the patient was recommended a conservative treatment. Both a CT scan of the chest and abdomen and a renal function test were performed on the patient on an annual basis as part of the follow-up period. Throughout the follow-up period, there was not a single episode of kidney-related issues.

## Discussion

A congenital defect in the diaphragm, BH, is a condition that allows herniation of the abdominal viscera to the thorax. BH is the most common type of CDH and is typically detected on the left side. This condition is attributed to the inability of pleuroperitoneal ducts to close completely at approximately the eighth week of pregnancy, together with the presence of primordial connections between the chest and abdomen [1]. This condition is commonly encountered in children and frequently causes symptoms in younger individuals. Infants tend to experience gastrointestinal symptoms, while children often have respiratory symptoms [1,6]. CDH can be detected during the prenatal period and in neonates using ultrasound, and its presence can be confirmed by CT or magnetic resonance imaging (MRI) scans. This is an extremely rare occurrence in adults, with fewer than 110 documented examples [1,8]. In adults, it typically does not show any symptoms and is frequently found during pre-surgical evaluations. Chronic symptoms are observed in symptomatic cases. The individual experiences gastrointestinal symptoms such as recurring abdominal pain, vomiting, or postprandial fullness, as well as respiratory symptoms, including cough, wheezing, difficulty breathing, and pain in the chest [8,9]. This abnormality is frequently observed on the left side, accounting for 80% of cases, while only 20% occur on the right side. BH, which affects both sides of the body, is extremely uncommon and frequently life-threatening. On the left side, omentum, large intestine, spleen, kidney, and pancreas are the organs that are most likely to herniate, whereas, on the right side, the liver is the organ that is most likely to herniate. BH has been documented as a cause of ectopic intra-thoracic kidney disease; however, this occurrence is extremely uncommon [1,10].

An ectopic kidney is a rare phenomenon that occurs in approximately one in 1,000 live newborns. Intra-thoracic ectopic kidney is a very rare condition, making up less than 5% of all ectopic kidney cases. Most of the cases are predominantly seen in males, with a male-to-female ratio of 2:1. Incidence of the intra-thoracic kidney that results from CDH is less than 0.25%, with the left side being mostly affected [1,11-14].

Specific structural characteristics that result from developmental rotation of the intra-thoracic kidney include more proximal origin of renal vessels, longer ureter, and posteriorly faced renal hilum. Despite the presence of these abnormalities, the intra-thoracic ectopic kidney usually functions normally [1,12-15]. In the current patient, the intra-thoracic ectopic kidney was found to be normal in size and showed normal attenuation and enhancement, with the contrast being promptly excreted into the pelvicalyceal system during CT urography.

Unlike other cases of intra-thoracic renal ectopias, the intra-thoracic kidney with BH is mobile and can be descended from thorax to retroperitoneal space. Most intra-thoracic kidneys do not show any symptoms, and surgery is only required if there is an obstruction in the ureter or vesicoureteral reflux. Surgery is the primary mode of action in symptomatic or complex cases, while conservative treatment is preferred for asymptomatic patients. The surgical procedure involves reducing the intra-thoracic kidney and closing the defect [1,12-15].

## Conclusions

Intra-thoracic ectopic kidney with a BH is extremely rare and is typically detected on the left side. The structure and function of intra-thoracic kidneys are typically normal in the majority of cases. When it comes to the clinical setting, the intra-thoracic kidney is a rather uncommon medical condition that causes a variety of concerns for doctors in terms of diagnosis and treatment responsibilities. To avoid unwanted image-guided biopsies and surgical procedures, it is imperative to look for intra-thoracic kidneys in patients presenting with a thoracic mass or an elevated hemi-diaphragm.
